# Influencing Side-Effects to Medicinal Treatments: A Systematic Review of Brief Psychological Interventions

**DOI:** 10.3389/fpsyt.2018.00775

**Published:** 2019-02-05

**Authors:** Rebecca K. Webster, G. James Rubin

**Affiliations:** ^1^Department of Psychological Medicine, Institute of Psychiatry, Psychology & Neuroscience, King's College London, London, United Kingdom; ^2^The National Institute for Health Research Health Protection Research Unit in Emergency Preparedness and Response, King's College London, London, United Kingdom

**Keywords:** review, side-effects, medicine, nocebo effect, interventions, side-effect information

## Abstract

**Background:** Nocebo effects contribute to a large proportion of the non-specific side-effects attributed to medications and are mainly generated through negative expectations. Previous reviews show that interventions designed to change participants' expectations have a small effect on pain experience. They are also effective in reducing side-effects caused by exposure to sham medications. To date, there has been no review of the influence of such interventions on symptoms attributed to real medicinal treatments.

**Objective:** To review studies using a randomized controlled design testing the effect of brief psychological interventions compared to usual practice on the side-effect experience to medicinal treatments in healthy volunteers and patients.

**Methods:** We searched Web of Science, Scopus, Medline, PsycINFO, PsycARTICLES, and Cochrane CENTRAL using search terms for randomized controlled trials along with “nocebo,” “placebo effect,” “medication,” “side-effects,” and associated terms. Studies were eligible if they studied a human population, used an active medicine, delivered a brief psychological intervention intended to influence side-effect reporting compared to usual care or no intervention, and used a randomized controlled design. Because of the heterogeneity of the literature we used a narrative synthesis and assessed evidence quality using the GRADE approach.

**Results:** Our database search and supplementary search of the reference sections of included studies retrieved 50,140 citations. After screening, full text review and manual reference searches, 27 studies were included. The quality of the studies and evidence was judged to be low. The strongest and most consistent effect came from omitting side-effect information, although surprisingly de-emphasizing side-effects did not affect side-effect reporting. Other techniques, including priming, distraction, and altering the perception of branding, produced mixed results.

**Conclusion:** Brief psychological interventions can influence side-effect reporting to active medications. Research is currently investigating new ways to de-emphasize side-effects whilst still upholding informed consent, but larger confirmatory trials with suitable control groups are needed. The literature in this area would be improved by more detailed reporting of studies.

## Introduction

Nocebo effects, sometimes dubbed the placebo effect's “evil twin,” are the experience of noxious symptoms in response to an inert exposure (1). Nocebo effects can also refer to negative clinical outcomes which are not attributable to the actual pharmacological or physiotherapeutic action of an intervention (2). It is estimated that between 38 and 100% of side-effects reported to drugs taken for a large range of medical conditions are related to the treatment context, rather than the active ingredients of the medication itself (3).

These nocebo-related side-effects are important, as they can affect a patient's well-being (4) and influence their decision as to whether to adhere to their treatment regimen (5, 6). For example adverse media coverage surrounding the safety of statins and their reported side-effects has resulted in around 2,00,000 patients who are no longer taking their statins as directed leading to a predicted increase of 2,000 cardiovascular events in the next decade (7). This is despite the fact that most of these side-effects are probably nocebo-related (8). Perhaps unsurprisingly, side-effects can also result in substantial additional health care costs in terms of additional primary care and hospital visits and also the cost of wasted medication due to non-adherence (9).

Of the multiple factors that may contribute to the development of nocebo effects, expectations of symptoms appear to be the main contributor. These can be generated through verbal and written suggestions about what symptoms to expect, be implied by the apparent dose of a drug, and be learnt through classical conditioning and social observation (10). Studies have used these psychological mechanisms as a means to alter peoples' experience of experimentally induced pain (11), as well as pain following acute medical procedures, such as injections (12) and surgery (13). These effects have been studied in multiple reviews, showing that brief psychological interventions designed to change expectation of pain following treatment have a small but reliable effect on relieving patients' pain compared to usual care (14–16).

However, to our knowledge, there has been no review of whether such interventions can alter patient experience ofside-effects to medicinal treatments. Although evidence demonstrates that such interventions can be effective in altering side-effects reported following exposure to inert substances (10), it is also important to assess if these effects can be transferred to clinical practice. We therefore set out to review studies using a randomized controlled design testing the effect of brief psychological interventions compared to usual practice on the side-effect experience to medicinal treatments in healthy volunteers and patients. To answer the question: can brief psychological interventions influence the side-effect experience to medications?

## Methods

Our reporting of this systematic review adheres to the standards for the Preferred Reporting Items for Systematic reviews and Meta-Analyses (17). The protocol for this review was prospectively registered on PROSPERO (CRD42018091903).

### Identification of Studies

We searched the following electronic databases with a predefined search strategy: Web of Science, Scopus, OvidSp (Medline, PsycINFO, and PsycARTICLES) and Cochrane CENTRAL. We included Web of Science and Scopus for their coverage of the sciences and social sciences. OvidSp was chosen for its coverage of journals chiefly in the area of health sciences, and also for its inclusion of the databases PsycINFO and PsycARTICLES. Cochrane CENTRAL was included due to its coverage of randomized controlled trials and because it includes records which are derived from other sources to the ones already chosen.

In preliminary work we tested a variety of search strategies in an effort to balance specificity and sensitivity. Our final search strategy used the recommended search terms to identify randomized controlled trials (18) along with the terms and associated words for “nocebo,” “placebo effect,” “medication,” and “side-effects.” We used separate search strategies for each of the databases as these needed to be modified due to differences in MeSH terms, boolean operators and wildcards. A copy the search strategy we used for Medline can be seen in the Supplementary Material.

### Review Process

The search was initially carried out on 22nd March 2018 and updated on 22nd June 2018 following the identification of a relevant study published between this time. The initial electronic searches were combined using EndNote and duplicates were identified and deleted. The titles and abstracts of citations were then screened for potential relevance. If relevance was not clear from the abstract, the study was taken forward to the full text review. All full text versions of papers that were potentially relevant were then screened in relation to the inclusion criteria. Papers that met the inclusion criteria had their reference sections manually searched for other studies that could be included.

### Selection Criteria

Studies were eligible for inclusion in this review if they met the criteria below.

#### Population

Human population (healthy volunteers, patients and children were allowed).

#### Exposure

Active medicinal treatment (i.e., contains a pharmacological agent), associated with side-effects.

#### Intervention

A brief, psychological intervention delivered in one session and that could be feasibly introduced within a single doctor-patient consultation or treatment appointment. By psychological we mean an intervention that targets certain psychological processes, such as cognitive expectations, attention or learning. Interventions requiring biological or chemical stimuli were excluded because these are not purely psychological. As we wanted to identify interventions that could be easily incorporated into clinical practice, in-depth psychological interventions, such as cognitive behavior therapy, mindfulness, relaxation training or guided imagery, or that consisted of intensive educational packages were excluded as these typically are not delivered in one session and often take place over the course of a treatment.

#### Comparator

Usual care. We excluded studies with control conditions involving a different type of intervention.

#### Outcome

We included studies with an outcome of side-effects measured via self-report or inferred through objective measures. We followed the NICE (19) definition of a side-effect as “An effect of a drug (or treatment or intervention) that is additional to the main intended effect. It could be good, bad or neutral, depending on the circumstances.” For some studies, e.g., those concerning infant experience to vaccinations, side-effects were measured within minutes of the procedure. We excluded these on the bases that the “side-effects” were presumably related to the insertion of the needle rather than the effects of the vaccine itself.

#### Study Design

Used an experimental design in which participants were randomized or quasi randomized to receive the intervention or the control condition.

#### Other Criteria

Published in the English language.

### Data Extraction

We extracted data from the final set of studies using a data extraction table developed for this systematic review. Data extracted included the study design and methodology, main demographics of participants, description of intervention and control conditions, side-effect measures, statistical approach and results. We also extracted details about the mode of the intervention, its content and duration.

### Quality Assessment

We assessed the quality of all eligible studies using the Cochrane Collaboration's Risk of Bias tool for randomized controlled trials (20).

### Data Synthesis and Analysis

Due to the heterogeneity in the interventions that we included and the way that side-effects were measured, scored and analyzed, we used a narrative synthesis to analyse the results. There is no general consensus on the best way to carry out a narrative synthesis for systematic reviews (21). As such we decided to use a weight of evidence approach by identifying the quality of evidence for each type of intervention reviewed. To do this we used the GRADE approach (22) which is a transparent framework used to grade the quality of evidence included in systematic reviews and the strength of recommendations.

## Results

### Search Results

**Figure 1:**
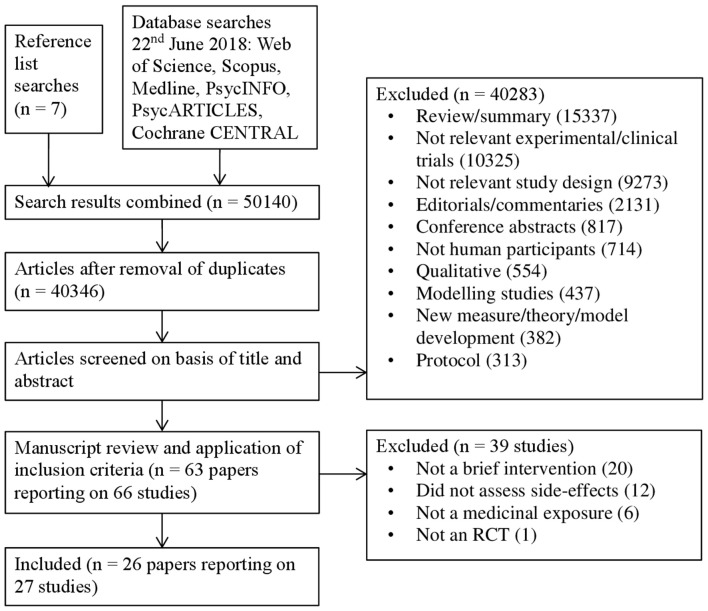
Flow diagram of the selection process of studies including the number of events and reasons for exclusion.

The database search retrieved 50,133 citations and searching the reference lists of included studies retrieved another 7, giving a total of 50,140. After removing duplicates 40,346 citations remained. After screening titles and abstracts, we reviewed the full text of 63 articles relating to 66 studies. Of these, 39 studies were excluded for not meeting the inclusion criteria, resulting in a total of 26 articles reporting on 27 studies. One article (23) reported results on two separate studies and is referred to in the text and tables as Study 1 or Study 2 where necessary. The number of studies at each stage of the search strategy and the reasons for exclusion are shown in Figure 1.

### Study Characteristics

See Table 1 for a full summary of the characteristics of the included studies. The 27 studies included in the review reported on a total of 3,459 participants. There was a range of patient groups and treatments under investigation. The most common of these were patients with cancer receiving chemotherapy (26, 31, 39, 41–44, 46), and patients with depression prescribed anti-depressants (29, 32, 35–37). All studies used a between participants RCT design apart from Cildag et al. (24), Myers and Calvert (36), Redd et al. (39), and Schagen et al. (42) which used a quasi-randomized approach, and Faasse et al. (28) who used a within-subjects RCT design. Some studies used a factorial design in their RCT involving different experimental conditions or baseline variables entered as independent factors (23, 31, 39, 41–43). In these cases, we have reported the main effects of the relevant intervention under investigation.

**Table 1:** Summary table of included studies.

**References (intervention type)**	**Design**	**Sample (N, M age, %Male)**	**Treatment**	**Experimental group (n)**	**Control group (n)**	**Side-effect measure (main outcome)**	**Timing of measure**
Cildag et al. (24) (Distraction)	Quasi RCT	Patients undergoing drug provocation test (112, 41.8, 32.1)	Various drug provocation tests	Kept busy filling questionnaires, arranging files and doing archiving (63)	Did not perform any tasks (49)	? (yes)	During provocation test
Cocco (25) (Omitting)	RCT	Patients with arterial hypertension (114, 57.7, 100)	Metroprolol	1. Metroprolol given under a code number with no information about active substance. Informed of all possible side-effects apart from erectile dysfunction (38) 2. Informed taking metroprolol and about all possible side-effects apart from erectile dysfunction (38)	Informed taking metroprolol and erectile dysfunction might occur (38)	IIEF (yes)	At the end of the 60-day trial
Colagiuri et al. (26) (Priming)	RCT	Patients undergoing chemotherapy (91, 60.5, 42.9)	Chemotherapy	Assessed expectancies for nausea, fatigue, sadness, and loss of appetite before first infusion (46)	No assessment of expectancies (45)	SSQ and EORTC QLQ-C30 (yes)	After first chemotherapy cycle
Colgan et al. (27) (Branding)	RCT	People with frequent headaches (69, 21, 21.7)	Branded and generic ibuprofen	Video interviewing health specialists and providing accurate information addressing misperceptions of generic medicines (34)	Video interviewing a neurologist about the different types of headaches and their global epidemiology (35)	Modified GASE (no)	An hour after taking ibuprofen for a headache
Faasse et al. (28) (Branding)	RCCT	Students with frequent headache (81, 20.8, 17)	Ibuprofen	Ibuprofen labeled with the brand name “nurofen” (81)	Ibuprofen labeled “generic ibuprofen” (81)	SSQ (yes)	An hour after taking ibuprofen for a headache
Faria et al. (29) (Misc)	RCT	Patients with seasonal affective disorder (46, 31.8, 60.9)	Escitalopram	Deceptively told they would receive the active placebo, likely to induce side-effects similar to escitalopram but out of which no symptom-improvement could be expected (22)	Correctly informed about the SSRI treatment and the expected improvement (24)	? (no)	?
Flam et al. (30) (Omitting)	RCT	Patients undergoing a myelogram (30, ?,43.3)	Myelogram	1. No tape-recorded message (10) 2. Tape-recorded message about the techniques performed during a myelogram (10)	Tape-recorded message about the sensations to expect during a myelogram (10)	Interview (no)	The day following the myelogram
Jacobs et al. (31) (Priming)	RCT	Patients with breast cancer (175, 49.4, 0)	Chemotherapy	1. Informed about the possibility of cognitive problems after chemotherapy (56) 2. Same information plus reassurance that there are still patients who score well on memory tests (59)	Received a one sentence neutral introduction without reference to chemotherapy or cognitive difficulties (60)	Cognitive failure questionnaire (yes)	After study introduction
John et al. (32) (Omitting)	RCT	Patients with depression (39, 34.0, 38)	Antidepressants	Asked about their symptoms, their prescription was explained to them and they were encouraged to take their medicines as advised (22)	Face-to-face, 10-min education session about depression, treatment, efficacy and adverse effects of the prescribed drug, and plan of management (17)	KAE questionnaire (no)	Six weeks after prescription
Lauder et al. (33) (De-emphasizing)	RCT	Patients undergoing hysterectomy (195, 43.4, 0)	Anesthesia	Informed of the use of two perioperative antiemetics to reduce the incidence of emetic symptoms to anesthesia after operation (95)	Informed that this was a study of post-operative well-being with no information about emetic symptoms (100)	Recorded by nursing staff (yes)	Immediately, 4, 8, and 24 h after surgery (combined)
Mondaini et al. (34) (Omitting)	RCT	Patients with benign prostatic hyperplasia (107, 60.5, 100)	Finasteride	Finasteride concealed as an “X compound of proven efficacy for the treatment of BPH” (52)	Finasteride concealed as an “X compound of proven efficacy for the treatment of BPH. It may cause erectile dysfunction, decrease libido, problems of ejaculation but it is uncommon” (55)	Male sexual function-4, and SSQ (yes)	12 months after prescription
Mukherjee and Sahay (23) Study 1 (Omitting)	RCT 2 × 2	Business graduates (117, ?, ?)	Skin cream	No information given. Price was manipulated at two levels: 1. low (?) 2. high (?)	Read a news item about skin creams and their potential side-effects. Price was manipulated at two levels: 1. low (?) 2. high (?)	SSQ (yes)	?
Mukherjee and Sahay (23) Study 2 (Omitting)	RCT 2 × 3	Business students (149, ?, ?)	Skin Cream	Presented with an excerpt devoid of any negative aspects. Price was manipulated at three levels 1. low (?) 2. high (?) 3. discounted (?)	Read a news item about skin creams and their potential side-effects. Price was manipulated at three levels 1. low (?) 2. high (?) 3. discounted (?)	SSQ (yes)	?
Myers and Calvert (35) (Omitting)	RCT	Patients with depression (93, 40.4, 39.8)	Amitriptyline	Told only that the drug was being given to cure their depression (46)	Told the drug was being given to cure their depression and listed a series of side-effect they might experience (47)	SSQ (yes)	Two weeks after initial dose
Myers and Calvert (36) (Omitting)	RCT	Patients with depression (89, 38.9, 33.7)	Dothiepin	Told only that the drug was being given to cure their depression (43)	Told the drug was being given to cure their depression and listed a series of side-effect they might experience (46)	SSQ (yes)	Two weeks after initial dose
Myers and Calvert (37) (Omitting)	RCT	Patients with depression (120, 43.3, 25.8)	Dothiepin	1. Told only that the drug was being given to treat their depression and received no written information (40) 2. Verbal and written information about beneficial effects (40)	Verbal and written information about side-effects (40)	SSQ (no)	3 and 6 weeks after prescription
O'Connor et al. (38) (De-emphasizing)	RCT	Patients eligible for flu vaccine (292, 52.5, 6.7)	Influenza vaccine	Described the percentage who remain influenza and side-effect free. Accompanied by flip charts and decision aid poster highlighting the key points (148)	Described the percentage who acquire influenza and vaccine side effects. Accompanied by flip charts and decision aid poster highlighting the key points (144)	SSQ (no)	3 days after vaccination
Redd et al. (39) (Distraction)	Quasi RCT	Pediatric patients undergoing chemotherapy (26, 14.0, 73.2)	Chemotherapy	Played from a choice of video games for 10 min (13)	Permitted access to toys, books, games, and television (13)	Visual analog scale (yes)	10 min later
Rickels et al. (40) (Misc)	RCT	Patients with psychiatric disorders (169, 31.9, 34.3)	Tranquilisers	Study doctor who was a psychiatrist that was drug “enthusastic” (?)	Study doctor who was a psychiatrist that was drug “skeptical” (?)	Recorded by doctor (no)	2 and 4 weeks after prescription (combined)
Roscoe et al. (41) (De-emphasizing)	RCT	Patients with breast cancer (53, 51.5, 0)	Chemotherapy	Handout showing positive interpretation of the data from two acupressure band studies. A 12 min CD involving visualizing pleasant scenes and explaining the efficacy of the acupressure bands (25)	Handout thanked patients for participating in the study, and the CD did not discuss the efficacy of the bands (28)	Diary (yes)	Each day for 5 days following chemotherapy (combined)
Schagen et al. (42) (Priming)	Quasi RCT 2 × 2 × 2	Patients with breast cancer (261, 53.7, 0)	Chemotherapy	Told that some patients treated with cytotoxic agents experience cognitive problems and the goal of the study was to obtain more insight into the relation between chemotherapy and cognitive problems' (130)	Received a neutral introduction (131)	SSQ (yes)	After study introduction
Schagen et al. (43) (Priming)	RCT 2 × 2	Patients with cancer (236, 48.4, 10.1)	Chemotherapy	Received the introduction that some patients treated with chemotherapy experience cognitive problems (116)	Received a neutral introduction (120)	Cognitive failure questionnaire (yes)	After study introduction
Shelke et al. (44) (De-emphasizing)	RCT	Patients undergoing chemotherapy (322, 57.6, 27)	Chemotherapy	Standard educational materials plus a handout explaining how effective ondansetron, would likely be in controlling nausea and vomiting (159)	Standard educational materials given to new patients (163)	SSQ (no)	Treatment day until four days after (combined)
Silvestri et al. (45) (Omitting)	RCT	Patients with newly diagnosed CVD (96, 52.0, 100)	Atenolol	1. Blinded to drug given (32) 2. Informed on drug given but not on the side-effects (32)	Informed on drug given and its side-effects on erectile function (32)	IIEF (yes)	After 3 months of treatment
Vasterling et al. (46) (Distraction)	RCT	Patients with cancer (60, 51, 35)	Chemotherapy	Played video games during chemotherapy treatment and told this would keep their minds off their treatment, making it less unpleasant and reducing the severity of its side effects (?)	Rested quietly in the treatment room before chemotherapy and told if a patient was relaxed, it would be less unpleasant, and the severity of the side-effects would be reduced (?)	SSQ (no)	After each of the 5 chemotherapy sessions (combined)
Wilhelm et al. (47) (De-emphasizing)	RCT	Healthy males (80, 24.5, 100)	Metoprolol	Informed of side-effects including headache, stomach pain or nausea. Dizziness is also mentioned, but is explained as a sign that the drug is starting to work and means that your body is responding to the beta-blocker particularly well (40)	Informed of side-effects including headache, stomach pain or nausea. Dizziness is also mentioned and explained as a potentially unpleasant, but already known side-effect of the drug (40)	Modified GASE (yes)	?
Wise et al. (48) (Omitting)	RCT	Patients with Asthma (237, 37, 23.8)	Montelukast	The computer presentation showed the same basic education but did not show positive messages about the expected benefits of montelukast and did not contain the television commercial. The capsules were off-white and referred to as montelukast (117)	Viewed a computer presentation for 10–20 min emphasizing the value and potency of the treatment including a television commercial for montelukast. The commercial also described potential side-effects. The capsules were 2-tone blue, and referred to as Singulair (120)	? (no)	After 2 and 4 weeks of treatment (combined)

*RCT, randomized controlled trial; RCCT, randomized controlled crossover trial; ?, not reported; CVD, cardiovascular disease; IIEF, international index of erectile dysfunction; SSQ, study specific questionnaire; EORTC QLQ-30, European Organization for Research and Treatment of Cancer Quality of Life Questionnaire; GASE, generic assessment of side effects scale; KAE, knowledge, attitudes, and experiences questionnaire*.

There were a variety of interventions used by included studies. We looked for common themes and content of the various interventions and were able to group the studies into five different types, these were: priming, distraction, branding, omitting side-effects, and de-emphasizing side-effects, plus a miscellaneous group.

The majority of studies used an un-validated questionnaire specifically designed for their study to measure side-effects, and measures were generally completed within days/weeks following treatment initiation.

### Quality Assessment

**Figure 2:**
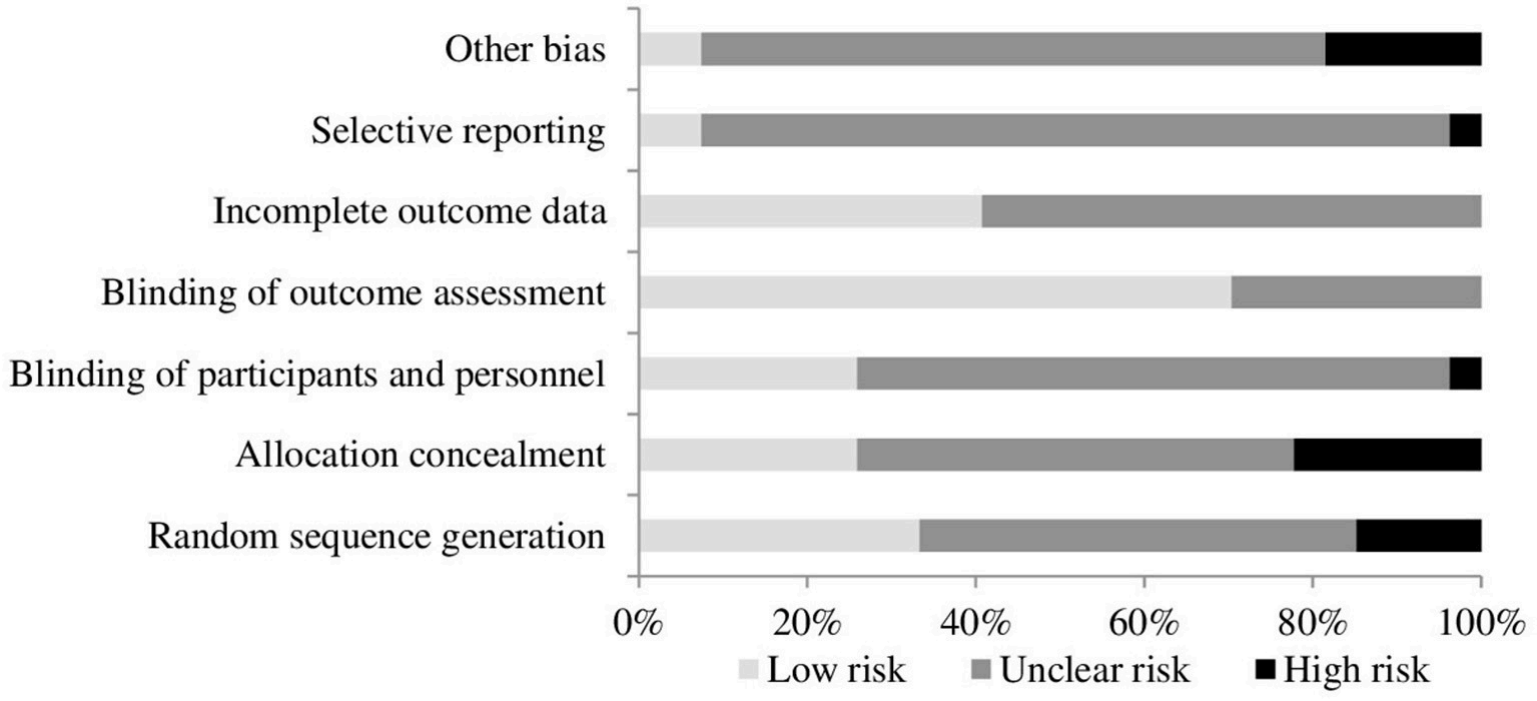
Quality assessment across included studies.

**Figure 3:**
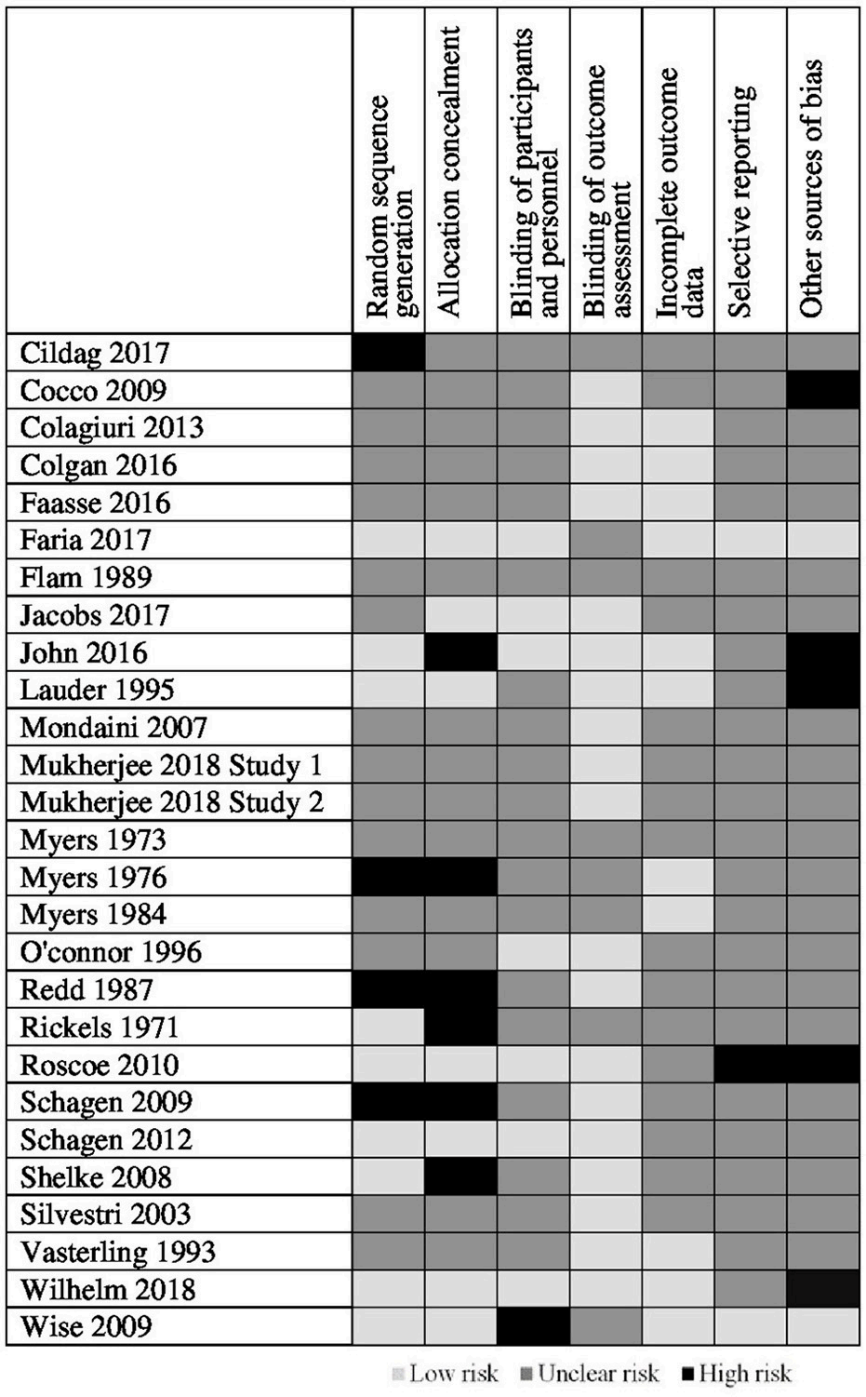
Quality assessment between included studies.

The quality of included studies was poor (see Figures 2, 3). The main problem was a lack of clear reporting within the papers. Over half of the studies neglected to mention how they carried out randomization, and four were at high risk for using a quasi-randomized approach. Because of the unclear reporting of random sequence generation, the risk for allocation concealment bias followed a similar pattern, and six studies were at high risk because their randomization approach allowed research staff to foresee subsequent allocations. For blinding of participants and personnel, studies often failed to state whether the experimenters were blind to the manipulation that accompanied the active treatment, leaving the risk of bias unclear. Only seven studies used adequate blinding procedures, with one not using blinding at all. Nineteen studies used side-effect measures which were completed by participants, as such blinding of the outcome assessment was judged unlikely to influence these results. For the remaining eight studies it was unclear if participants filled in the measures themselves or if they were administered by a blind/non-blind member of the study team. For 16 studies, drop outs were not addressed, or if they were, the paper typically failed to explain how this affected the results, leaving the risk of bias unclear; the remaining 11 studies provided adequate information and reasoning behind drop outs. Only two studies had lodged a protocol in a publicly accessible registry before the start of recruitment, leaving us unable to assess the risk for selective reporting for the remaining studies, apart from one in which there was a change in the prespecified primary analysis suggesting there was a high risk of bias.

### Quality of the Evidence

The quality of evidence regarding priming, distraction, omitting side-effect information and de-emphasizing side effects, and doctor characteristic intervention(s) was very low. This is because most of the information came from studies at low or unclear risk of bias, in which plausible bias could alter the results. There was also some evidence of inconsistency and imprecision in the results due to opposite findings, wide confidence intervals and some small studies which may not have been adequately powered. Due to the broad nature of this systematic review, there is no evidence of indirectness, as all included studies helped to answer the question. It is plausible however, there may have been some publication bias due to the preponderance of smaller studies.

The quality of evidence regarding the branding intervention studies was low. This was graded similarly due to the reasons discussed for the above interventions, however the inconsistency in the results could perhaps be explained by differences in the interventions, and we judged that the small studies were probably due to this literature representing an early evidence base, rather than publication bias.

Finally, the quality of evidence for the deception intervention was moderate. There was some imprecision evident and the sample size was small, however the study was judged to have a low overall risk of bias, and there was no evidence of indirectness. As only one study was included, inconsistency and publication bias could not be determined.

### Effect of Interventions on Side-Effect Reporting

#### Priming

Four studies looked at the effect of priming on side-effect reporting following chemotherapy with mixed results (see Table 2). Colagiuri et al. (26) found a slight trend for priming patients by assessing their expectancies for side-effects vs. no assessment on subsequent nausea. Jacobs et al. (31) and Schagen et al. (42) found no indication of an effect of priming patients by mentioning that chemotherapy is associated with cognitive problems on retrospectively reported cognitive side-effects. However, Schagen et al. (43) in a similar study did find a small effect of priming leading to increased reporting of previous cognitive side-effects to chemotherapy compared to those in a control group who received no such information.

**Table 2:** Priming intervention results.

**References**	**Side-effect outcome and analysis**	**Results**	**Effect size (95% CI)**	**Evidence quality**
Colagiuri et al. (26)	Occurrence: Multiple logistic regression	Nausea: Ns, priming group > control group, *p* = 0.06	OR = 3.19 (0.95, 10.69)	+ Very Low
		Fatigue, sadness, loss of appetite: Ns, lowest *p* = 0.31	–	
	Severity: Multiple linear regression	Nausea, fatigue, sadness, loss of appetite: Ns, lowest *p* = 0.24	–	
Jacobs et al. (31)	Frequency: 2 × 2 ANCOVA-group and chemotherapy experience as independent factors	Cognitive problems: Ns for those with chemotherapy experience	–	
Schagen et al. (42)	Severity: 2 × 2 × 2 ANOVA-group, pre-existing knowledge, and chemotherapy experience as independent factors	Cognitive problems: Ns, priming group (M = 2.53, SE = 0.11) < control group (M = 2.68, SE = 0.12) for those with chemotherapy experience, *p* = 0.34	d = −0.11 (−0.36, 0.13)	
		Other complaints: Ns, priming group (M = 3.00, SE = 0.09) > control group (M = 2.95, SE = 0.10) for those with chemotherapy experience, *p* = 0.75	d = 0.05 (−0.20, 0.29)	
Schagen et al. (43)	Severity: 2 × 2 ANOVA-group and chemotherapy experience as independent factors	Cognitive problems: Priming group (M = 21.20, SD = 6.4) > control group (M = 18.98, SD = 6.7) for those with chemotherapy experience, *p* = 0.032	d = 0.34 (0.08, 0.60)	

*Ns, non-significant; M, mean; SE, standard error; SD, standard deviation; OR, odds ratio; d, Cohen's d; –, insufficient detail to calculate effect size*.

#### Distraction

Three studies looked at the effect of distraction on side-effect reporting following chemotherapy and drug provocation tests, showing some evidence that distraction can reduce side-effect reporting (see Table 3). Cildag et al. (24) found that keeping patients busy with filling/archiving files significantly reduced the occurrence of adverse reactions compared to a control group, but only by a small amount. Redd et al. (39) found that distracting pediatric cancer patients with video games significantly reduced chemotherapy nausea from baseline, compared to those in the control group. However, for adult cancer patients, Vasterling et al. (46) found that video games were not effective in reducing chemotherapy nausea or vomiting compared to a control group.

**Table 3:** Distraction intervention results.

**References**	**Side-effect outcome and analysis**	**Results**	**Effect size (95% CI)**	**Evidence quality**
Cildag et al. (24)	Occurrence: Chi square	Adverse reaction: Distraction group (7.9%) < control group (34.7%), *p* = 0.0004	OR = 0.16 (0.05, 0.48)	+ Very Low
Redd et al. (39)	Severity: Repeated measures ANOVA-group as an independent factor and time as a within-groups factor	Nausea: Distraction group (M decrease = −16.92, SD = 8.70), *p* < 0.001. Control group (M decrease = −1.77, SD = 8.96), *p* > 0.05	–	
Vasterling et al. (46)	Frequency: Univariate analysis	Vomiting: Ns	–	
	Severity: Univariate analysis	Nausea: Ns	–	

*Ns, non-significant; M, mean; OR, odds ratio; –, insufficient detail to calculate effect size*.

#### Branding

Two studies looked at the effect of branding on side-effect reporting to ibuprofen showing some evidence that branding can affect side-effect reporting (see Table 4). Colgan et al. (27) found that a video designed to correct participants' beliefs about generic medicines significantly reduced side effects for both branded and generic ibuprofen compared to those in a control group, showing a large effect. However, Faasse et al. (28) found that simply changing the labeling of ibuprofen from branded to generic did not significantly affect side-effect reporting.

**Table 4:** Branding intervention results.

**References**	**Side-effect outcome and analysis**	**Results**	**Effect size (95% CI)**	**Evidence quality**
Colgan et al. (27)	Frequency: Linear mixed models	Side-effects across both types of ibuprofen: Generic medicines video group (M = 1.18, SE = 0.40) < control group (M = 2.57, SE = 0.39), *p* = 0.02	d = −0.68 (−1.17, −0.20)	++ Low
		Side-effects within each type of ibuprofen: Generic medicines video group < control group, for both branded, *p* = 0.02, and generic, *p* = 0.035	–	
Faasse et al. (28)	Severity: Linear mixed models	Side-effects: Ns, branded (M = 3.41, SE = 0.47) > generic (M = 2.95, SE = 0.46) ibuprofen, *p* = 0.16.	–	

*Ns, non-significant; M, mean; SE, standard error; d, Cohen's d; –, insufficient detail to calculate effect size*.

#### Omitting Side-Effect Information

Eleven studies looked at the effect of omitting side-effect information on side-effect reporting to a range of different treatments, showing that omitting side-effects significantly decreases side-effect reporting (see Table 5). Eight studies found that not informing patients about potential side-effects significantly decreased side-effect reporting to metropolol (25), a myelogram (30), antidepressants (32), finasteride (34), skin cream (23) (study 1 and 2), atenolol (45), and montekulast (48) compared to a control group which received side-effect information, each showing large effect sizes. Similarly Myers and Calvert (36) found a trend for a decrease in side-effect reporting when patients were not informed about the side-effects to the antidepressant dothiepin compared to a control group, and Myers and Calvert (37) found that side-effects significantly decreased to dothiepin when comparing the group that only received beneficial information to groups that received no information and side-effect information. Only one study, Myers and Calvert (35), found no effect of side-effect information on subsequent side-effect reporting.

**Table 5:** Omitting side-effect information intervention results.

**References**	**Side-effect outcome and analysis**	**Results**	**Effect size (95% CI)**	**Evidence quality**
Cocco (25)	Occurrence: Chi square	Erectile dysfunction: No side-effect information group (8%) < drug information group (13%) < control group (32%), *p* < 0.01	–	+ Very Low
Flam et al. (30)	Frequency: Independent t test	Side-effects: Procedural information group (M = 2.1) < control group (M = 4.3), *p* < 0.05	d = −0.94 (−1.86, −0.02)	
		Side-effects: No side-effect information group (M = 1.6) < control group (M = 4.3), *p* < 0.05	d = −1.23 (−2.19, −0.27)	
John et al. (32)	Frequency: Mann Whitney U	Side-effects: No side-effect information group (M = 1.7, SD = 1.9) < control group (M = 3.5, SD = 2.6), *p* = 0.044	d = −0.81 (−1.47, −0.15)	
Mondaini et al. (34)	Occurrence: Mann Whitney U	One or more side-effects: No side-effect information group (15.3%) < control group (43.6%), *p* = 0.03	OR = 0.23 (0.09, 0.59)	
		Erectile dysfunction: No side-effect information group (9.6%) < control group (30.9%), *p* = 0.02	OR = 0.24 (0.08, 0.70)	
		Decreased libido: No side-effect information group (7.7%) < control group (23.6%), *p* = 0.04	OR = 0.27 (0.08, 0.89)	
		Ejaculation disorders: Ns, No side-effect information group (5.7%) < control group (16.3%), *p* = 0.06	OR = 0.31 (0.08, 1.23)	
Mukherjee and Sahay (23) study 1	Severity: Two-way ANCOVA with group and price as independent factors	Skin dryness: No side-effect information group < control group, *p* = 0.01	ηp^2^ = 0.05	
		Skin dryness: Ns difference between pricing, *p* > 0.70	–	
		Skin dryness: Interaction between pricing and side-effect information, *p* = 0.02. No side-effect information group (M = 1.85, SD = 1.38) < control group (M = 3.39, SD = 1.98) at low price. Ns between no side-effect information group (M = 2.51, SD = 1.09) and control group (M = 2.72, SD = 1.51) at high price	ηp^2^ = 0.04	
Mukherjee and Sahay (23) study 2	Severity: Two-way ANCOVA with group and price as independent factors	Skin dryness: No side-effect information group < control group, *p* = 0.01	ηp^2^ = 0.04	
		Skin dryness: Ns difference between pricing, *p* = 0.32	–	
		Skin dryness: Ns interaction between pricing and side-effect information, *p* = 0.19	–	
Myers and Calvert (35)	Occurrence: Chi square	Side-effects: Ns, no side-effect information group (73.9%) < control group (80.9%), *p* > 0.05	OR = 0.67 (0.25, 1.79)	
Myers and Calvert (36)	Occurrence: Chi square	Side-effects: Ns, no side-effect information group (51.2%) < control group (71.7%) *p* > 0.05	OR = 0.41 (0.17, 1.00)	
Myers and Calvert (37)	Occurrence: ?	Side-effects at 3 weeks: Ns, no side-effect information group (67.7%), beneficial information group (48.4%), control group (73.7%), *p* > 0.05	–	
		Side-effects at 6 weeks: Ns, %), no side-effect information group (63.6%), beneficial information group (29.6), control group (57.1%), *p* > 0.05	–	
		Side-effects at 3 weeks: Beneficial information group (48.4%) < combined no side-effect information group and control group (70.5%), *p* < 0.05	OR = 0.39 (0.16, 0.96)	
		Side-effects at 6 weeks: Beneficial information group (29.6%) < combined no side-effect information group and control group (60.0%), *p* < 0.05	OR = 0.28 (0.10, 0.76)	
Silvestri et al. (45)	Occurrence: Chi square	Erectile dysfunction: Blind to drug group (3.1%) < drug information group (15.6%) < control group (31.2%), *p* < 0.01	–	
Wise et al. (48)	Occurrence: Logistic regression	Headaches: No side-effect information group (29%) < control group (37%), *p* = 0.02.	–	
		Lethargy, gastrointestinal distress, fever, rhinitis, cough, “flu,” and skin rash: Ns	–	

*Ns, non-significant; M, mean, SD, standard deviation; OR, odds ratio; d, Cohen's d; ηp^2^, partial eta squared; –, insufficient detail to calculate effect size; ?, not reported*.

#### De-emphasizing Side-Effects

Five studies looked at the effect of de-emphasisng side-effects on side-effect reporting to range of different treatments, showing evidence that this seems to have no effect (see Table 6). Three studies found that informing patients of side-effects but in a way that does not make them seem as bad had no effect on side-effect reporting to anesthesia (33), or chemotherapy (41, 44), however this was compared to a control group that did not receive any information about side-effects. O'Connor et al. (38) found that positively framing side-effects to emphasize those that remain side-effect free and comparing to a control group that received standard information about side-effects significantly reduced side-effect reporting to the flu vaccine. Wilhelm et al. (47) found that positively framing side-effects by explaining they are a sign that the drug is working did not significantly reduce side-effects to metoprolol compared to those who received standard information.

**Table 6:** De-emphasizing side-effects intervention results.

**References**	**Side-effect outcome and analysis**	**Results**	**Effect size (95% CI)**	**Evidence quality**
Lauder et al. (33)	Occurrence: Chi square	Vomiting: Ns, positive suggestion group (40.4%) < control group (46.9%), *p* = 0.363	OR = 0.77 (0.43, 1.36)	+ Very Low
		Nausea: Ns, positive suggestion group (63.4%) < control group (73.2%), *p* = 0.148	OR = 0.64 (0.34, 1.18)	
	Severity: Mann Whitney U	Nausea: Ns, positive suggestion group (M = 1.45, SD = 1.88) < control group (M = 1.80, SD = 1.89), *p* = 0.087	d = −0.19 (−0.50, 0.13)	
O'Connor et al. (38)	Occurrence: Chi square	Sore arm, weakness, fever: Ns	–	
		Myalgia: Positive frame group < control group, *p* = 0.01	–	
		Chills: Positive frame group < control group, *p* = 0.003	–	
Roscoe et al. (41)	Severity: Two-way ANCOVA with group and baseline expectancy as two independent factors	Average nausea: Ns, positive suggestion group (M = 1.94, SD = 1.07) < control group (M = 1.96, SD = 0.99), *p* > 0.05	d = −0.02 (−0.56, 0.52)	
		Peak nausea: Ns, positive suggestion group (M = 3.56, SD = 2.09) < control group (M = 3.57, SD = 1.88), *p* > 0.05	d = −0.01 (−0.54, 0.53)	
Shelke et al. (44)	Occurrence: Chi square	Nausea: Ns, positive suggestion group (79%) > control group (73%), *p* = 0.19	OR = 1.41 (0.84, 2.37)	
	Severity: Independent t test	Nausea: Ns, positive suggestion group (M = 1.86, SE = 0.76) > control group (M = 1.76, SE = 0.76), *p* = 0.34	d = 0.01 (−0.21, 0.23)	
Wilhelm et al. (47)	Frequency: Independent t test	Specific side-effects: Ns, positive suggestion group (M = 1.38, SD = 1.56) < control group (M = 1.75, SD = 1.77), *p* = 0.318	d = −0.22 (−0.66, 0.22)	
		Nonspecific side-effects: Ns, positive suggestion group (M = 0.68, SD = 1.02) < control group (M = 1.15, SD = 1.93), *p* = 0.174	d = −0.30 (−0.75, 0.14)	
	Severity: Independent t test	Specific side-effects: Ns, positive suggestion group (M = 1.60, SD = 2.00) < control group (M = 1.85, SD = 2.02), *p* = 0.580	d = −0.12 (−0.56, 0.31)	
		Nonspecific side-effects: Ns, positive suggestion group (M = 0.83, SD = 1.60) < control group (M = 1.30, SD = 3.12), *p* = 0.396	d = −0.20 (−0.63, 0.25)	

*Ns, non-significant; M, mean; SE, standard error; SD, standard deviation; OR, odds ratio; d, Cohen's d; –, insufficient detail to calculate effect size*.

#### Other Interventions

Two other studies investigated interventions which do not fall into the above categories (see Table 7). Faria et al. (29) deceptively told seasonal affective disorder patients that they would receive an active placebo which would produce similar side-effects to escitalopram when in fact they received the active drug itself and found this showed a trend in decreasing reported side-effects compared to a control group who were correctly informed. Rickels et al. (40) found no effect of the prescribing psychiatrist being a drug “enthusiast” or drug “skeptical” on reported side-effects to tranquilisers among psychiatric patients.

**Table 7:** Miscellaneous results.

**References**	**Side-effect outcome and analysis**	**Results**	**Effect size (95% CI)**	**Evidence quality**
Rickels et al. (40)	Occurrence: ?	Side-effects: Ns	–	+ Very Low
Faria et al. (29)	Frequency: Independent t test	All side-effects: Ns, *p* = 0.17	–	+++Moderate
		Drug related side-effects: Ns, covert group (M = 2.22, SD = 1.38) < control group (M = 3.39, SD = 2.62), *p* = 0.06	d = −0.55 (−1.14, 0.04)	

*Ns, non-significant; M, mean; SD, standard deviation; d, Cohen's d; –, insufficient detail to calculate effect size; ?, not reported*.

## Discussion

### Summary of Main Findings

Although previous literature has looked at altering side-effects generated in response to inert exposures, it is important to test if these interventions also work in the clinical setting and affect side-effects to real medications which may be initiated or exacerbated through a nocebo effect. This can then provide the basis for introducing into clinical practice strategies to reduce these side-effects. Unfortunately, the quality of the studies identified in this review were generally low quality mainly due to the lack of clear reporting, inadequate randomization and allocation procedures, and unpowered effects. Our overriding recommendation, therefore, is that additional, better quality work is needed in this field.

This point notwithstanding, from the results of the included studies, the strongest and most consistent effect in altering side-effects experienced following medical treatments was omitting information about side-effects. Other techniques, such as priming, distraction and altering the perceptions of branding produced mixed results. More tentatively, studies which investigated over the counter medications, common prescription medications, and vaccines seemed to be more susceptible to these interventions than those which studied chemotherapy.

The finding that omitting side-effect information produced the most consistent and strongest effect supports the evidence from the literature on inert exposures (10) which recommends that in order to reduce side-effects induced by nocebo effects we should avoid giving suggestions of side-effects associated with medications to patients. It also echoes what is found in experimental nocebo studies which find that altering information about side-effects alters side-effect experience to infrasound (49), and electrical pain stimuli (50). In addition, this supports previous work showing that interventions designed to change patients' expectations of pain by altering verbal suggestions about the pain to expect after a treatment or procedure can relieve (placebo) or increase (nocebo) patients baseline pain depending on the suggestion (15, 16), highlighting the role that expectations play in both placebo and nocebo effects. Perhaps unsurprisingly no study looked at the effect of omitting information about side-effects to chemotherapy, and therefore we cannot say if the results extend to chemotherapy too. However, as chemotherapy is already well-known for its side-effects, it may be that omitting side-effect information would do little to alter subsequent side-effect reporting in this group.

Not mentioning side-effects to patients in order to reduce these effects is ethically problematic and may not meet the requirements of informed consent, something which has been widely discussed in the literature (51, 52). An alternative approach is to explain the potential side-effects to patients in a way that de-emphasizes them and reduces their apparent likelihood or severity (53). At first look, the results of studies which have used this approach do not appear promising. Most studies have showed no effect of de-emphasizing side-effects on subsequent side-effect experience. However, this might be an artifact relating to the design of these studies, in which the groups that received the de-emphasized side-effect information were compared to a control groups that received no side-effect information at all. Explaining side-effects to patients, albeit in a positive light, is still likely to increase the perceived likelihood of side-effects compared to not describing side-effects. It would be interesting for future studies to test the effects of de-emphasizing side-effects of medication compared to a suitable control group which receives standard side-effect information. In other studies which used an appropriate control group, positive framing of side-effects was shown to be beneficial, a finding that has also been reproduced in healthy adults taking an inert tablet (54). There is also scope for further investigations about framing the side-effects of medication as a sign that the drug is working. This was investigated in a pilot study that, although not powered to find an effect, nonetheless showed a decrease in side-effect measures among participants who believed the medicine to be harmful (47). This idea of de-emphasizing side-effects has shown some promise in the placebo literature on pain, in which positive messages which focus more on the beneficial outcome of treatments rather than the potential side-effects may be more effective in relieving patients pain compared to usual care messages (14).

Priming patients by informing them about the side-effects to chemotherapy and then asking them to recall side-effects, or by asking about their expectations of chemotherapy side-effects overall showed little impact on side-effect reporting. This may be due to the treatment under investigation. Chemotherapy is a high-profile treatment, and as such it is likely patients are already aware of the side-effects that accompany it, limiting the effect that priming could have. In experimental studies, priming patients using pain-related fear has been shown to increase sensitivity to heat stimuli (55). It may be that priming patients about side-effects to lower profile drugs find more promising effects.

Distraction techniques have been shown to be effective in the field of pain research for example experimental and needle-related pain (56, 57), but in terms of medication side-effects, the evidence base is not as large, limiting conclusions. From the results, it seems that distraction tasks should be relevant to the patients to have the greatest chance of being effective. For example, while video games are suitable for reducing side-effects to chemotherapy in pediatric patients they are less effective in adults (39, 46).

The effect of branding on side-effects shows some effect, something also reflected in the inert literature (58). However given the early evidence base, future studies are needed to test the effects of branding on prescribed drugs, and interventions to alter patients' perceptions of prescribed generic drugs.

### Quality of Original Research

It is possible that some of our conclusions may be due to differences in quality between those studies that found an effect and those that did not. We did not observe any clear trend for lower quality studies to report more or fewer significant results than higher quality studies. However, overall the quality of the studies included in this review was limited due to poor reporting of key issues in experimental research, such as randomization, allocation concealment, blinding, and not registering a study protocol prior to initiating recruitment. In addition, the quality of evidence from these studies was low, partially due to these risk of bias issues, but also the fact that the samples sizes of studies were relatively small, adding to evidence of imprecision and indirectness due to the wide confidence intervals, and sometimes contradictory findings.

### Quality of This Review

Search strategies for systematic reviews based on nocebo effects are difficult to balance in terms of their specificity and sensitivity (10). In this instance we deliberately opted for a broad search strategy in order to identify as many relevant studies as possible. Due to time constraints, screening, data extraction and quality assessment were done by primarily one author. However, there were regular weekly meetings with both authors to discuss screening, data extraction, quality assessment and writing up of the results, allowing us to resolve any issues as they arose.

Other limitations of the review reflect the way we grouped the results. We aggregated studies based on the type of intervention under investigation. These groupings contained different side-effect outcomes, treatments and participants. It is possible that interactions exist between these variables and the interventions under investigation. Unfortunately, due to the small number of studies investigating each intervention, we did not have enough data to explore this in any depth. However, it does appear that chemotherapy might not be as susceptible to brief psychological interventions compared to prescription and over-the-counter drugs.

### Implications and Future Directions

Not mentioning potential side-effects to patients has the most consistent effect in reducing side-effects to medical treatments, especially for over-the-counter and prescription drugs. Whether this meets ethical or regulatory requirements is debatable, however (53, 59). De-emphasizing side-effects through positive framing has potential and could be introduced within doctor-patient consultations and in accompanying patient information leaflets for patients to read at home. Further testing of this method especially in terms of reframing side-effects as signs that the drug is working is needed in an adequately powered trial. In addition, it is important for future studies testing ways of de-emphasizing side-effects to adequately compare them to a control group that receives the standard side-effect information.

Besides framing, it is also important for doctors to consider patients beliefs about generic medicines if prescribing generic drugs or switching patients from a previously branded medication to a generic. Colgan et al. (27) suggest that a simple explanation of how the pharmacological ingredients in generic drugs do not actually differ with branded drugs would be useful. So far, the effects of branding have been studied in over the counter and inert tablets: research with prescribed medication is now needed. In addition, distraction could be beneficial for use if age appropriate tasks are used.

Finally, only one study investigated the effect of doctor characteristics on side-effects. This represents a surprising gap in the literature. Doctor characteristics, such as empathy have been shown to be important in benefitting patients for a range of clinical conditions, especially pain (60). We believe this is an important avenue for future research to investigate in terms of benefitting patients by reducing medication side-effects.

## Conclusion

This review was restricted by the quality and heterogeneity of the included studies, limiting the conclusions that can be drawn. It does however, provide an indication of which brief psychological interventions are effective in reducing side-effects to active medical treatment. The clearest effect was from omitting information about side-effects to participants before exposure. Although withholding side-effect information would be one way to reduce this, it is ethically and legally problematic. Current work is looking at how we can effectively de-emphasize side-effects while still giving patients the information needed for informed consent, and this shows promise. Potential strategies include positively framing the risk of side-effects, focusing more on the benefits of the drug, and framing side-effects in terms of signs that the drug is working. However further research is needed in larger trials with suitable control groups. There is also a gap for future research to consider doctor characteristics, such as empathy, as a means of reducing patients' experience of side-effects. Finally, better reporting of studies is essential in future, allowing for more concrete determinations of study quality.

## Author Contributions

RKW and GJR developed the initial research question for the systematic review and the search strategy. RKW carried out the search, screening, data extraction and quality assessment with regular input from GJR. RKW wrote the first draft which was subsequently revised by GJR.

### Conflict of Interest Statement

The authors declare that the research was conducted in the absence of any commercial or financial relationships that could be construed as a potential conflict of interest.
