# Supplementary material for: Influencing Side-Effects to Medicinal Treatments: A Systematic Review of Brief Psychological Interventions
Source: Front Psychiatry. 2019 Feb 5;9:775. doi: 10.3389/fpsyt.2018.00775 (PMC6371736; doi:10.3389/fpsyt.2018.00775)
Supplement: Supplementary file 1 [file Table_1.docx]

MEDLINE (via OVID)

| 1 | randomized controlled trial.pt. |
| --- | --- |
| 2 | controlled clinical trial.pt. |
| 3 | Randomized Controlled Trials/ |
| 4 | Random Allocation/ |
| 5 | Double-Blind Method/ |
| 6 | Single-Blind Method/ |
| 7 | or/1-6 |
| 8 | Animal/ not Human/ |
| 9 | 7 not 8 |
| 10 | clinical trial.pt. |
| 11 | exp Clinical Trial/ |
| 12 | (clinic* adj25 trial*).tw. |
| 13 | ((singl* or doubl* or trebl* or tripl*) adj (mask* or blind*)).tw. |
| 14 | Placebos/ |
| 15 | placebo*.tw. |
| 16 | random*.tw. |
| 17 | Research Design/ |
| 18 | (latin adj square).tw. |
| 19 | or/10-18 |
| 20 | 19 not 8 |
| 21 | 20 not 9 |
| 22 | Comparative Study/ |
| 23 | exp Evaluation Studies/ |
| 24 | Follow-Up Studies/ |
| 25 | Prospective Studies/ |
| 26 | (control* or prospectiv* or volunteer*).tw. |
| 27 | Cross-Over Studies/ |
| 28 | or/22-27 |
| 29 | 28 not 8 |
| 30 | 29 not (9 or 21) |
| 31 | 9 or 21 or 30 |
| 32 | (nocebo* or expectancy or expectation* or conditioning or conditioned or social observ* or social model* or social learn* or ((placebo or context* or nondrug or non-drug or nonpharmacological or non-pharmacological or nonspecific or non-specific or situational) adj (action* or effect* or influence* or intervention* or response* or result*))).mp. or ASSOCIATION LEARNING/ or “Conditioning (Psychology)”/ or SOCIAL LEARNING/ or Nocebo Effect/ or Placebo Effect/ |
| 33 | (side effect* or (adverse adj1 event*) or adverse effect* or (adverse adj1 reaction*) or (symptom* adj15 attribut*)).mp. or "Drug-Related Side Effects and Adverse Reactions"/ |
| 34 | (medic* or treatment* or topical or inhal* or intranasal* or rectal* or drug* or tablet* or pill* or capsule* or vaccin* or immuni#* or innoculat* or inject*).mp. or MEDICINE/ or Drug Therapy/ or Pharmaceutical Preparations/ or Vaccines/ or Immunization/ or Injections/ |
| 35 | 31 and 32 and 33 and 34 |
